# Congenital Hypothyroidism and the Deleterious Effects on Auditory Function and Language Skills: A Narrative Review

**DOI:** 10.3389/fendo.2021.671784

**Published:** 2021-08-10

**Authors:** Caio Leônidas Oliveira Andrade, Crésio de Aragão Dantas Alves, Helton Estrela Ramos

**Affiliations:** ^1^Department of Life Sciences, University of the State of Bahia, Salvador, Brazil; ^2^Medical School, Institute of Health Science-Federal University of Bahia, Salvador, Brazil; ^3^Post-Graduate Program in Medicine and Health, Medical School of Medicine, Federal University of Bahia, Salvador, Brazil; ^4^Postgraduate Program in Interactive Processes of Organs and Systems, Health & Science Institute, Federal University of Bahia, Salvador, Brazil; ^5^Bioregulation Department, Health and Science Institute, Federal University of Bahia, Salvador, Brazil

**Keywords:** thyroid, congenital hypothyroidism, hearing loss, auditory system, hypothyroidism

## Abstract

Congenital hypothyroidism (CH) is an endocrine disease commonly found in newborns and is related to the absence or reduction of thyroid hormones (THs), which are essential for development since intrauterine life. Children with CH can develop hearing problems as THs are crucial for the auditory pathway’s development and maturation. Sensory deprivations, especially in hearing disorders at early ages of development, can impair language skills, literacy, and behavioral, cognitive, social, and psychosocial development. In this review we describe clinical and molecular aspects linking CH and hearing loss.

## Introduction

The development of auditory pathways depends on the presence of adequate serum levels of thyroid hormones (TH) and their action on TH receptors (1–3). These hormones regulate proteins and enzymes responsible for the structural formation of the inner ear, being crucial for the proper performance of auditory function (4).

In fact, TH deficiency or TH defect action can cause severe changes in the development of the auditory system (2, 5). Clinical situations of reduction or absence of normal serum levels of TH, such as congenital hypothyroidism (CH), are frequently associated with hearing loss (6, 7). However, the incidence of hearing loss (HL) in individuals with CH is still uncertain, and it could affect ~20% of patients (8–10), occurring isolated or associated with vertigo and tinnitus (11).

It is well-known that when sensory deprivation events occur in the first months of life, a period considered critical for the maturation of biological functions, there is a high potential for subsequent significant delays in language, cognition, academic, emotional, and social development (12, 13). Therefore, the early detection and intervention of hearing problems, even in subclinical stages, allow individuals with auditory dysfunction to obtain sociolinguistic performances close to normal hearing (12, 14).

Given these facts and considering the scarcity of literature on the subject, the present study sought to achieve a narrative review on the probable dysfunctions of the auditory pathways connected to CH and TH deprivation in early neonatatal period, and its adverse impacts on social performance and language acquisition and development.

### Clinical Aspects of Congenital

CH is an endocrine disease commonly found in newborns (15) with a worldwide occurrence of approximately 1:2,000 to 1:4,000 live births. It mainly affects females in the proportion of roughly 2:1 (16). CH is a public health issue, which can be detected with newborn screening (NS). The lack of an early diagnosis and adequate treatment can result in neurological and motor development changes and irreversible mental retardation (17). The main objective of neonatal screening is to promote the detection of congenital diseases before the symptomatic phase, enabling early treatment (18). CH does not usually present symptoms at birth, or there are only subtle manifestations of the disease, making clinical diagnosis difficult (19). The neonatal screening program recommends a TSH cut-off level of 10 mUI/L (7, 20). Newborns with high TSH in the test are referred for evaluation and confirmation of the diagnosis (21). Confirmation of the diagnosis of CH is made with laboratory exams, showing TSH greater than 15 mUI/L and total or free T_4_ with normal or low values (7, 17).

CH is normally classified as permanent or transient, whose etiology is classified into primary, secondary, and tertiary. The permanent condition requires lifelong treatment, as the hormonal deficiency is persistent. On the other hand, transient CH regains typical TH production in the initial months or years of life. In permanent primary cases, thyroid dysgenesis (TD) corresponds to 85% of cases, whereas dyshormonogenesis (DH) represents 10 to 15% of cases (22). In secondary CH, the lesion is in the pituitary; and in the tertiary form, the dysfunction is in the hypothalamus. The last two cases are extremely rare. Central CH (secondary or tertiary) is commonly associated with other pituitary hormonal deficiencies (15, 17). The absence of stimuli from pituitary TSH (Thyroid Stimulating Hormone) or hypothalamic TRH (Thyrotropin-Releasing Hormone) is the cause of deficient hormone production in the central CH (23).

The etiology and clinical phenotype of CH become essential in determining the severity, outcomes, and treatment of the disease, as patients may need therapy with higher doses and close monitoring, especially during early periods of life (24). THs are essential for adequate neurodevelopment since intrauterine life (25). Their absence leads to dysfunction of specific brain areas, affecting regions such as the posterior parietal, inferior temporal lobes, caudate nucleus, and hippocampus, which are responsible for, respectively, spatial location, object identification, attention, and memory (26, 27).

### Action of Thyroid Hormones on Auditory Function

THs play an essential role in developing the inner ear during the embryonic period (28). Since the fetal period, the T_3_ is essential for auditory development, when the embryo in the first trimester depends exclusively on maternal THs, beginning its hormonal synthesis in the second half of the gestational period (2, 3). Triiodothyronine (T_3_) is mediated by the thyroid hormone receptor (THR), whose action on cochlear sensory cells is caused by the differential expression of thyroid hormone receptor alpha (THRα) and thyroid hormone receptor beta (THRβ) (21).

In the rodent cochlea, THRs are expressed in the sensory epithelium and other tissues from mid-gestation into the postnatal period and function as transcription factors playing important roles in control target genes relevant for auditory development and function, and the abnormal regulation of genes controlled by THRs has been assumed to be the origin of neurosensory deafness associated with CH (29).

The *THRα* gene is widely expressed throughout the spiral organ of corti, while the *THRβ* gene has its expression prominently in the greater epithelial ridges of sensitive hair cells (5, 30, 31). This gene expression pattern points out that the spiral organ is a direct-action site for TH and explains the scientific evidence of morphological and functional abnormalities of the structures that form the cochlea in cases of hypothyroidism (28, 32–37). Indeed, the THRs’ expression is timely coordinated in order to have a very precise signaling necessary for proper THR-dependent differentiation events, comprising complete inner sulcus, sensory epithelium, spiral ganglion, cochlea, and auditory nerve maturation (38).

**Table 1** summarizes mouse models of TH action or production defects. Actually, THRα1 is considered non-essential for hearing, while defects on THRβ, in mice, present deafness linked to cochlear alterations. On animal models, THRβ-null mice show threshold elevations ranged from a few decibels to complete loss of auditory responsiveness. An isoform-specific importance ranking is observed, because only THRβ1 signaling defect is associated with retardation in the expression of the fast-activating potassium conductance in inner hair cells, whereas deletion of the THRβ2 isoform does not lead to anormal cochlear function (38).

**Table 1:** Mouse models for understanding the relevance of genes involved in thyroid development, hormone biosynthesis, and thyroid hormone action on hearing function.

Gene	Molecular mechanism	Thyroid phenotype	Hearing function
*Pax8* ^–/–^	Inactivation of the *Pax8* gene	CH, Athyreosis	Deafness, degeneration of outer hair cells Deafness, sensorineural hearing loss
*Tshr^hyt/hyt^*	Autosomal recessive mutation in the TSHR gene	CH, Thyroid hypoplasia	Deaf-mutism, abnormality in the outer hair cell morphology Deafness, sensorineural hearing loss
*TRβ^–/–^*	Inactivation of the *TR*β gene	Resistance to thyroid hormone	Deafness, sensorineural hearing loss
*TRβ^tm1/tm1–^*	*TR*β gene point mutation reducing the affinity of TR to TH		Deafness
*TRα1^–/–^β^–/–^*	Compound *TRα1 and β genes*	Resistance to thyroid hormone	Deafness
SECISBP2	Gene indirectly disrupt T3 signaling by inhibiting translation of deiodinases		Hearing loss Otitis media
SLC26A4	Gene codifica o transportador de ânions Pendrin.	Goiter Pendred syndrome Defective organification of iodide in the thyroid gland	Non-syndromic deafness Sensorineural hearing loss Enlarged vestibular aqueduct in the inner ear
*DIO 2 ^-/-^*	Deletion of Dio2		Deafness

PAX8, paired box gene 8; SECISBP2, selenocysteine-insertion sequence binding protein 2; TSHR, thyroid stimulating hormone receptor (TSHR); DIO2, type 2 deiodinase; THRα, thyroid hormone receptor alpha; THRβ, thyroid hormone receptor beta; CH, congenital hypothyroidism; SLC26A4, solute carrier family 26 member 4.

Nonetheless, deletion of both THRβ1 and THRα1 produces exacerbated defects that simulate those provoked by hypothyroidism (38). In reality, human genetic alterations associated with loss of TRβ function, a condition named resistance to TH, also result in deafness (39).

The critical developmental time period of the middle and inner ears occurs in parallel to the natural elevation of TH serum plasma levels. Thyroxine (T_4_), liberated by the thyroid gland into the circulation, must be metabolically activated or inactivated by iodothyronine deiodinases, and 3,5,3’-triiodothyronine (T_3_) is the main ligand of the THRs. Therefore, TH adequate intracellular levels are accomplished after action of deiodinase type 2 (D2) and deiodinase type 1 (DI) encoded by Dio2 and Dio1, respectively (29).

**Figure 1:**
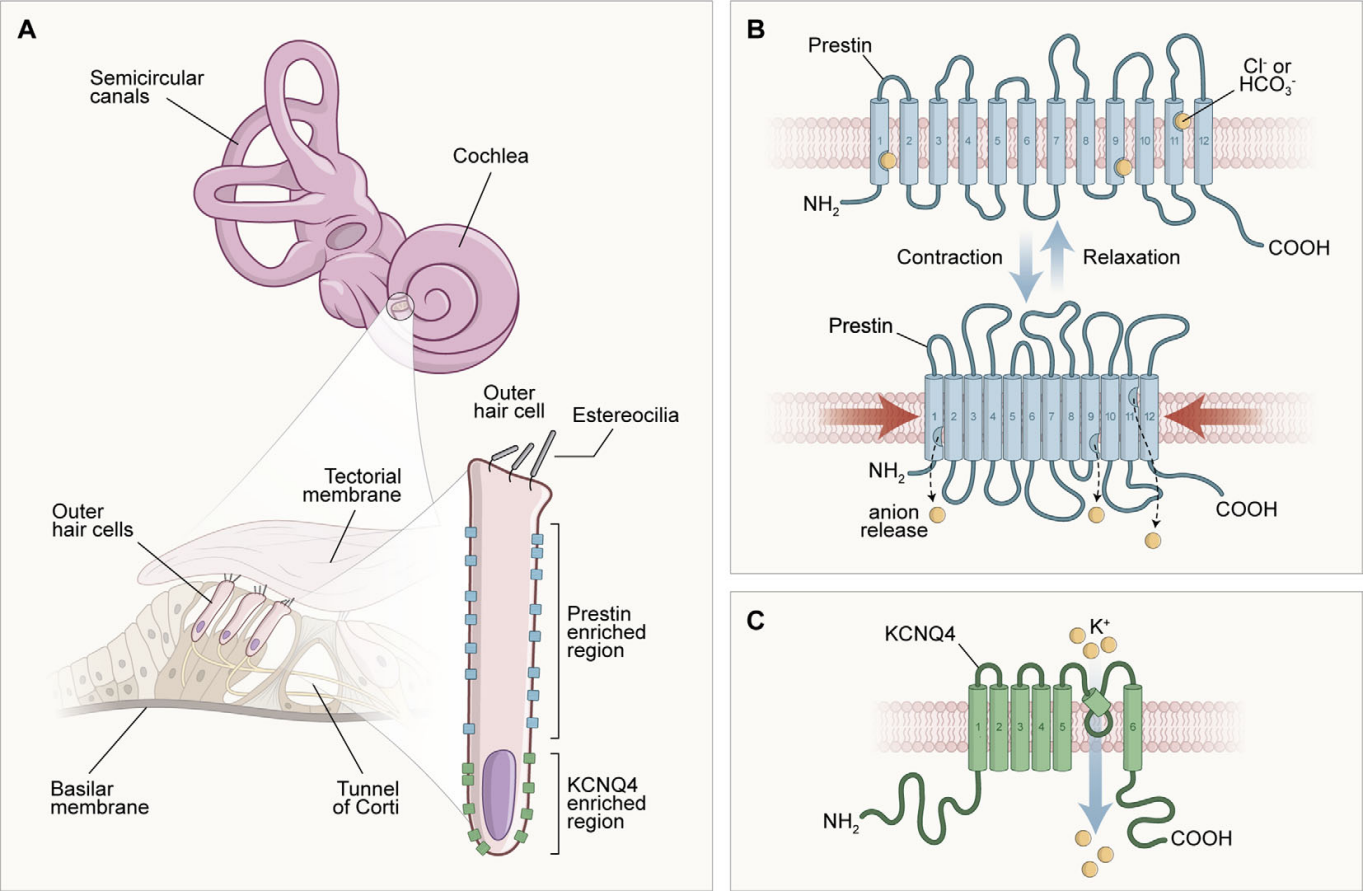
Scheme of the morphological configuration and special distribution of transmembrane proteins responsible for the endolymphatic potential (KCNQ4) generation and cochlear electromotility (prestin). In **(A)**, the structures of the inner ear are illustrated in different parts: at the top of the image, there are the cochlea and semicircular canals, structures that compose the inner ear macroscopically; to the left of the image, in a microscopic analysis, there is the spiral organ (Tunnel of Corti) formed of the tectorial membrane, supporting and sensory cells; and to the right of the image, the morphology of the outer hair cell can be observed, delimiting the molecular location of the transmembrane proteins prestin (lateral) and KCNQ4 (basal). In **(B)**, it is possible to observe the unique arrangement of the prestin protein and its physiology. Prestin is involved with the motor function of outer hair cells (OHC). This activity occurs when OHCs are depolarized through the influx of K+ positive electrical charges after a sound stimulation, creating a positive intracellular environment that favors the displacement of Cl- anions from the prestin's binding sites into the cytoplasm. This electrical charge movement causes a shortening of prestin and, consequently, a reduction in the size of the OHC, characterizing the active mechanism and the bio-electromotility of the OHC that occurs in the absence of calcium and ATP. In **(C)**, after depolarization, an OHC enters the repolarization and hyperpolarization stage due to the exit of cations in the basal portion of the potassium channels formed by the transmembrane protein KCNQ4, which contributes to the generation of the endolymphatic potential.

Other evidence that suggests strong influences of TH in the cochlea is related to the expression of the *SLC26a5* gene, which encodes the prestin protein. This protein is considered the outer hair cells (OHC) engine in the cochlear amplification process (40), which is reduced, immature, and with reduced distribution under hypothyroidism conditions (41–43). The gene expression encoding the K^+^ channels, *KCNQ4*, responsible for the endolymphatic potential formation, has also been discussed in the literature. Therefore, it has been shown that these ion channels are significantly reduced and poorly distributed under conditions of thyroid hypofunction (44). **Figure 1** illustrates the molecular structures inherent in external hair cells, which are dependent on adequate serum levels for thyroid hormones in the body.

Hormone deficiency can cause reductions in β-tectorin protein in the tectorial membrane, which explains structural abnormalities of the tectorial membrane and cochlear function. The OHCs are susceptible to serum thyroid hormone levels (36). Thus, serum levels of thyroid hormones circulating in the bloodstream can affect cell differentiation, which reduces the amount of organelles in the cytoplasm (37). These changes may be accompanied by abnormalities of the afferent dendrites and delayed growth of the efferent terminals that make direct connections with the OHC (4).

In the case of neural and central structures, studies in animal models have shown abnormalities in myelination and reduction of axons in the anterior commissure and corpus callosum (36). There is also a reduction in the number of microtubules in the neural cytoplasm and an altered distribution of apical dendrites of the pyramidal neurons (37). Added to this, there are records of a decline in the deoxyglucose levels of the metabolism marker in regions such as the cochlear nucleus, superior olivary complex, nuclei of the lateral lemniscus, inferior colliculus, medial geniculate body, and auditory cortex. Therefore, it is possible to state that TH deficiency will significantly affect the auditory pathway (42).

### Consequences of CH on Auditory System

Hearing is one of the essential senses for human communication, and it is where the individual develops speech. In addition, it is through hearing that the process of acquisition and development of oral language occurs. The auditory system consists of a peripheral portion (outer, middle, and inner ears), which captures sounds and transforms them into electrical impulses, and a central portion (brain auditory pathways), responsible for the analysis and interpretation of what is heard (45). Any complication in one of these portions can result in hearing loss (46), compromising not only communication but also receptive and expressive language, literacy, school performance, and the child’s psychosocial development (45).

The functionality of the thyroid gland is crucial for the development of the auditory system (1). THs are vital for auditory pathway morphogenesis and maturation (3), and the deficiency of these hormones jeopardizes the development of hearing (47). Therefore, CH can result in hearing loss (27), and even with early treatment, small hearing changes can be observed in individuals with CH (48). This happens due to the cochlea’s susceptibility to metabolic disorders, resulting from its intense activity and low energy reserve (49). THs act in both systems (peripheral and central) in the auditory system, and they are responsible for forming key structures of the inner ear, such as the cochlear duct, organ of Corti, and tectorial membrane (50). Therefore, the shortage or lack of THs brings losses to these structures.

Audiological changes noted in CH patients are diverse. However, losses with sensorineural, bilateral, and symmetrical characteristics are often found, with degrees varying from mild to moderate (8, 9). Actually, the risk of hearing loss may be associated with the severity of CH (43). In the researched literature, hearing changes in CH are characterized as peripheral or central, of insidious occurrence, with impaired auditory abilities (cognitive functions related to hearing). These skills are essential to the development of oral and written language and social-emotional progress. Additionally, they affect the individual in periods considered critical to developing global skills and full stage of experimentation and interaction with the environment, compromising the quality of life. In this context, when a hearing disorder is detected early, even during the neonatal period, early intervention through speech therapy and indication of hearing aids, if necessary, may be required and performed, preventing future harm to the child.

### Impact of Hearing Loss at Early Ages

In cases where TH deficiency occurs in early periods, as in CH, the risk of hearing loss in children is increased (8, 10). This data is significantly worrying when thinking about the harm that the reduction or absence of action of TH in the crucial periods of neurological development and maturation can bring. The central nervous system is one of the most affected (51), and it can alter the processing of the acoustic signal up to the cortex, causing difficulties in auditory skills (52) that will result in problems with behavioral, language, and social difficulties.

The crucial periods for the development of children’s hearing and oral language occur in early childhood. Nerve structures are already specialized in the brain of newborns with auditory cortical areas formed and ready to receive acoustic stimuli from the external environment. Consequently, the first contact with sounds is provided, instigating the mother tongue’s acquisition and increasing the linguistic repertoire (53, 54). Hence, when newborns have alterations in their auditory pathways that limit them to having an adequate sound sensation during the first 3 years of life, their linguistic and social potential will be low and reduced (53, 54). In the absence or deficit of sound stimuli at critical times, without adequate intervention, the child may present vital educational, social, and emotional delays (12).

The literature also shows that some language deficits, fine motor skills, visuospatial processing, attention and memory, and hearing disorders can persist in patients with CH even with early treatment (47, 48). Even moderate or mild hearing loss can alter the hearing perception of voiceless phonemes (55, 56), making the understanding of soft speech unintelligible, even in a quiet environment (57). As a result, phonological discrimination, phonological awareness, and phonological memory are compromised, consequently interfering in the learning processes of these children, directly affecting their quality of life and their families (58, 59).

## Conclusion

THs are essential for brain and intellectual development, as well as for peripheral and central auditory functions that extend from the fetal period to 2 years of age, a period considered critical for typical development. Therefore, CH can be considered a potential risk factor for changes in acoustic signals’ processing mechanisms along the auditory pathway, which manifests itself as cognitive, language, and socioemotional delays.

## Author Contributions

CLOA: conception, writing. CA: Editing, review. HR: Conception, writing. All authors contributed to the article and approved the submitted version.

## Conflict of Interest

The authors declare that the research was conducted in the absence of any commercial or financial relationships that could be construed as a potential conflict of interest.

## Publisher’s Note

All claims expressed in this article are solely those of the authors and do not necessarily represent those of their affiliated organizations, or those of the publisher, the editors and the reviewers. Any product that may be evaluated in this article, or claim that may be made by its manufacturer, is not guaranteed or endorsed by the publisher.

## Abbreviations

CH, congenital hypothyroidism; T_4_, Hormone thyroxine; hypothalamic TRH, Thyrotropin-Releasing Hormone; NNSP, National Neonatal Screening Program; NS, Newborn screening; PAX8, Paired box gene 8; *SECISBP2*, Selenocysteine-insertion sequence binding protein 2; T_3_, Triiodothyronine; TH, thyroid hormones; TSH, Thyroid Stimulating Hormone; TR, Thyroid hormone receptor; *THRα*, thyroid hormone receptor alpha; THRβ, Thyroid hormone receptor beta; TSHR, Thyroid Stimulating Hormone receptor; DIO2, Type 2 deiodinase; OHC, Outer hair cells.
